# Difficulties encountered removing locked plates

**DOI:** 10.1308/10.1308/003588412X13373405386411

**Published:** 2012-10

**Authors:** S Raja, AM Imbuldeniya, Garg S, G Groom

**Affiliations:** ^1^James Paget University Hospitals NHS Foundation Trust,UK; ^2^West Hertfordshire Hospitals NHS Trust,UK; ^3^King’s College Hospital NHS Foundation Trust,UK

**Keywords:** Locked plates, Less invasive stabilisation system, Locking compression plate, Hardware removal, Complications, Cold welding

## Abstract

**INTRODUCTION:**

Locked plates are commonly used to obtain fixation in periarticular and comminuted fractures. Their use has also gained popularity in repairing fractures in osteoporotic bone. These plates provide stable fixation and promote biological healing. Over the last 3 years, we have used over 150 locked plates with varying success to fix periarticular fractures involving mainly the knee and ankle. In this study, we report our clinical experience and the difficulties encountered when removing locked plates in adult patients with a variety of indications including implant failure, infection, non-union and a palpable symptomatic implant.

**METHODS:**

A retrospective analysis was performed of patients enrolled prospectively into a database. Included in the study were 36 consecutive adult patients who each underwent the procedure of locked plate removal in a single inner city level 1 trauma centre. Data collected included primary indication for fixation, indication for implant removal, time of the implant in situ, grade of operating surgeon and difficulties encountered during the procedure.

**RESULTS:**

Implant removal was associated with a complication rate of 47%. The major problems encountered were difficulty in removing the locked screws and the implant itself. A total of ten cold welded screws were found in eight cases. Removal was facilitated by high speed metal cutting burrs and screw removal sets in all but one case, where a decision was made to leave the plate in situ.

**CONCLUSIONS:**

The majority of studies investigating implant removal and problems encountered in doing so report a relatively high complication rate. With the advent of locking plates and their growing popularity, difficulties are now being seen intra-operatively when removing them. There is a paucity of data, however, specifically directed at locking plate removal. We recommend that surgeons should be aware of the potential complications while removing locked plates. Fluoroscopic control and all available extra equipment (mainly metal cutting burrs and screw removal sets) should be available in theatre.

Implant removal is a common orthopaedic procedure and consumes considerable resources.^1^ The clinical indications for implant removal are not well established and the procedure itself can be quite challenging, with associated complications. Removal of conventional plates, intramedullary nails, tension band wires and screws have been reported to be associated with complication rates of up to 40%.^2^ Most of these complications are implant or surgeon related. Commonly, the most junior member of the team is given responsibility for implant removal at the end of the operating list. It has been shown that the rate of complication is related directly to the level of the surgeon’s experience.^2–4^

Recently, more sophisticated implants have been made available to the orthopaedic community, which has added further demands to the technique of implant removal. Additions have included the locking compression plate (LCP), the less invasive stabilisation system (LISS) and the Proximal Humeral Interlocking System (PHILOS; Synthes, Welwyn Garden City, UK). Advantages of these locked internal fixators have been seen for periarticular and comminuted fractures as well as fixation in osteoporotic bone.^5^

Recent literature, however, suggests that fracture fixation with these plates can be complicated by implant failure, malunion, non-union, infection and a steep learning curve.^6–8^ These implants, like any other, may require removal. To date, there have been isolated reports of difficulties encountered during removal of LISS plates.^9,10^ We describe our experiences and problems encountered during the removal of locked plates from a consecutive series of 36 patients.

## Methods

Between July 2004 and October 2007, 36 patients aged between 23 and 90 years were admitted to hospital for locked plate removal. The data were obtained retrospectively from case notes and peri-operative radiographs. All patients operated on during this period were included and there were no exclusions. The indications for implant removal were clear in all of the cases and were identified by the senior author. Data collected from each case included the patient’s age, sex, date of index operation, indication for surgery, type of implant, indication for implant removal, date of implant removal, level of operating surgeon and any difficulties encountered.

All operations were performed on an inpatient basis under regional or general anaesthesia. A full open surgical approach was used in each case as difficulty was encountered in removing these implants through mini-incisions. Fluoroscopy guidance was used in all cases. As suggested by the manufacturer, the implant removal kit was used in each case to remove the implant although some cases required additional instrumentation. Post-operatively, patients were followed up in the outpatient department for a mean of six weeks.

## Results

Overall, 36 locked plates were listed for removal from 36 consecutive patients. There were 17 male and 19 female patients with an average age of 48.4 years (range: 23–90 years). The implants were removed between 5 and 588 days after insertion. The mean time that the metal had been in situ was 507.8 days. The most common indication for implant removal was a painful or palpable implant in 17 patients, followed by fracture non-union in 9. Implant failure and avascular necrosis accounted for three cases each while two locked plates were removed in response to patient demand. A further two plates were removed to help facilitate another procedure. Infection accounted for a single case.

Thirteen implants were removed by trainee surgeons, of which there were two cases in which complications arose. The remaining 23 implants were removed by senior surgeons with complications occurring in 15 cases, hence demonstrating a complication rate of 47% in this series. Further breakdown revealed that 15% was from trainees and 65% from senior surgeons.

**Figure 1:**
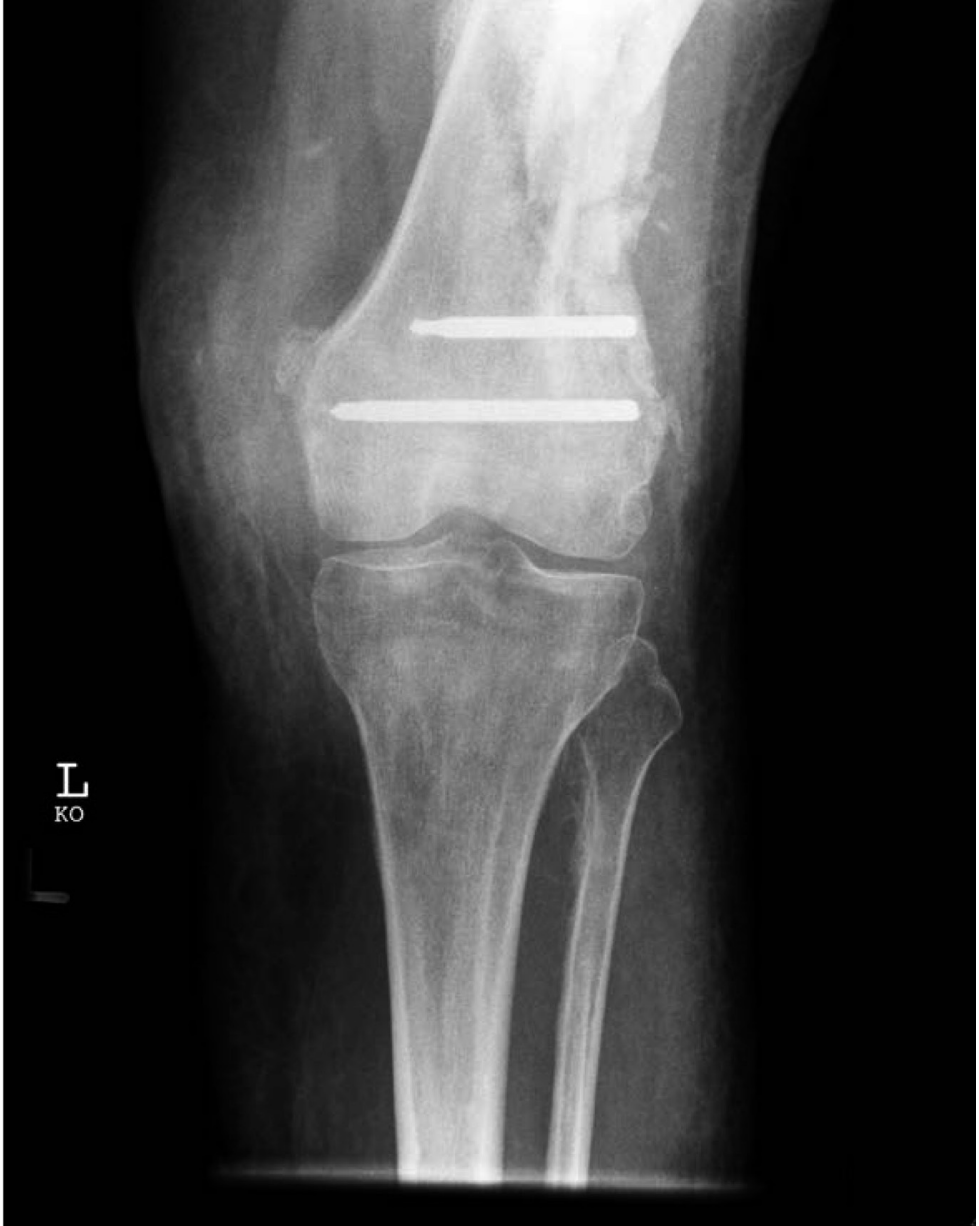
Damaged locking screws left in distal femur from the less invasive stabilisation system plate (heads destroyed with high speed cutting burr)

The most frequent problem encountered was cold welding and stripping of screws. Ten screws in eight patients were found to be cold welded to the plate (Table 1). They could not be removed from the plate and in one case the plate had to be removed with the screw still cold welded to it (Fig 1). One screw head was found to be stripped. Six plates (1 distal femoral LISS, 2 distal tibial LISS, 1 proximal tibial LISS, 1 femoral LCP and 1 PHILOS) were found broken, along with two broken screws from a separate distal femoral LISS plate. Three patients, whose indications for removal were non-union, had further treatment by refixation and bone grafting.

**Table 1:** Implant used and number of cases in which removal of locked plate was complicated

	Proximal tibial LISS	Distal tibial LISS	Distal tibial LCP	Distal femoral LISS	Distal femoral LCP	PHILOS	Total
**Total number of implants**	9	3	2	7	3	12	**36**
**Complications**							
Cold welding	5	0	1	1	0	1	**8**
Broken plate	1	2	0	1	1	1	**6**
Broken screw	0	0	0	1	0	0	**1**
Infection	1	0	0	0	0	0	**1**
Continued pain	0	0	0	1	0	0	**1**

LISS = less invasive stabilisation system; LCP = locking compression plate; PHILOS = Proximal Humeral Interlocking System

In all cases difficulty arose trying to remove screws that were engaged tightly with the plate. The Midas Rex® (Medtronic, Fort Worth, TX, US) pneumatic high speed metal cutting burr was used to cut the metal plate around the stripped screw hole to facilitate removal of the plate. Once the plate was removed, the screws were removed. The Synthes screw removal set was used in four cases to facilitate screw removal. These techniques are not always successful and in one case the procedure was abandoned and the plate was left in situ.

## Discussion

The clinical indications for implant removal are not well established and there are no definitive guidelines. In a study based on 5,095 implant removals, 80% of all internal fixation devices were reported to be removed.^1^ It was concluded that without a strict implant removal policy, a remarkable portion of the resources allocated for elective orthopaedic operations (29% in this series) was spent on routine hardware removal procedures.

Indications for implant removal are currently based on the clinician’s personal preferences and the patient’s demands. The most common reported indications for implant removal have been pain perceived to be coming directly from the implant, irritation of surrounding soft tissues and implant failure. There have also been reports of metal allergy, metal detection, cosmesis and carcinogenesis.^3,6,7,9,10^ Local osteopaenia, stress fractures, metal toxicity, corrosion and malignancy have been reported to be associated with retained metal implants.^11–13^ However, the current literature does not support routine removal of implants to protect against allergy, carcinogenesis or metal detection.^14–16^ Nevertheless, it is clear that in a situation where the implant has failed or is infected, it needs to be removed.

The complications associated with implant removal are well documented.^2–4,6,9,10,17–19^ Most importantly they include infection, damage to neurovascular structures and wound healing. An anatomical study performed on cadavers concluded that there was a significant risk to the superficial peroneal nerve when using the distal 3 holes on a 13-hole distal tibial LISS plate.^20^ This danger was further exposed by Langkamer and Ackroyd.^2^ They described a 40% complication rate following implant removal with 16 out of 22 of these complications being sensory losses secondary to nerve damage. They concluded that unless absolutely necessary, implant removal should not be undertaken.

Sanderson *et al* reported an overall complication rate of 20% in a series of 188 patients who had implants removed.^3^ In this series, the highest complication rate (42%) was seen in forearm implant removals with the main complications being infection and nerve palsy. Beaupre *et al* reported refracture rates of up to 21% following removal of 459 plates from the forearm.^18^ In another study looking at implant removal in patients treated for slipped upper femoral epiphyses, at least one screw had to be left in situ in five out of the six patients involved.^21^ However, in a 2007 study involving 57 patients, no complications were reported following implant removal.^22^ The conclusion from this study was that following fracture healing, removal of hardware is safe with minimal risk and that improvement in pain relief and function can be expected.

Very little has been written in the literature about the removal of locked plates.^9,10^ Georgiadis *et al* reported the removal of three LISS plates and reported 17% of the screws were stripped at the time of removal.^10^ They had to cut the LISS plate in all three patients despite removing three screws successfully with a conical screw extraction device. They used a carbide-tipped metal cutting burr to destroy the plate. The indications for plate removal were pain and subcutaneous irritation of the implant. In another case report, Button *et al* reported removal of four LISS plates due to plate breakage and failure.^7^ Cole *et al* removed 4 out of 77 locked plates used to fix proximal tibia fractures.^23^ These authors did not specify any difficulty in removing these plates although they commented that plates were found to be loose at the time of removal.

Appropriate pre-operative planning allows for removal of tightly engaged stripped screws in most cases. During this study, a generous incision over the previous scar was found to be useful and availability of fluoroscopy in theatre mandatory. The Midas Rex® pneumatic high speed metal cutting burr was a highly useful part of the implant removal kit. It was used to cut the metal plate around the stripped screw hole to facilitate removal of the plate or to destroy the interface between the threaded screw head and plate hole. Once the plate was removed, the screws were either removed from the bone with the torque screwdriver or the Synthes screw removal set, or left in situ. Another reported method for removing stripped screws not used in this study is the foil interposition technique.^24^

There are a number of different reasons why locking screws may be difficult to remove. The anatomically shaped plates are on occasion further contoured intra-operatively by the surgeon. If bent at the level of the screw hole, this can cause a mismatch in engagement between the locking threads on the screw head and those in the plate hole. Malalignment of more than 5° between the threads can cause the screw to become loose and fail. This can also cause stripping of the screw heads if the screw is not centred in the hole, which is difficult to avoid when using a minimally invasive technique.^8^

The importance of tightening screws with the supplied torque limiting screwdriver, which limits the tightening torque to 1.5Nm, should not be underestimated. Overtightened screws or cross-threading can cause deformation of the screw heads and contribute to cold welding.^25^

Cold welding occurs when reactive metal surfaces come into close contact and lose their oxide layer. These apparently smooth metal surfaces are rough on a molecular scale. The protuberant features of each surface are called asperities. True contact between two such metal surfaces occurs at points where asperities come together forming asperity junctions. Only a very small proportion of the apparent contact area between metal surfaces is therefore actually in contact. Under high pressure at these points, contacting metal will adhere.^26^ Newer locking plates and screws available on the market are fully surface coated using the Dotize® system (Dot, Rostock, Germany). This aims to suppress protein adsorption, reducing the tendency towards cold welding. The implication is that major implant manufacturers are already aware of problems similar to those described in our study.

Finally, it is known that removal of metalwork is usually performed by the most junior member of the team.^4^ It has been reported that the rate of complication following implant removal increases if performed by a junior surgeon.^2,3^ We suggest that the operating surgeon should have appropriate experience of using locking plates before attempting their removal. It is also advised that the surgeon informs patients about implant removal and associated complications when obtaining informed consent for internal fixation.

Although our results displayed a higher rate of difficulty when senior surgeons were involved, it is likely that more complicated cases were being allocated to them pre-operatively. These results instead demonstrate how difficult removal of implants can be, even in the hands of experienced surgeons, and that to try and avoid the high difficulty rates (such as 47%) associated with this locked plate removal study, it is advised that senior surgeons remove the majority of these plates with all the appropriate equipment available to hand.

## Conclusions

**Figure 2:**
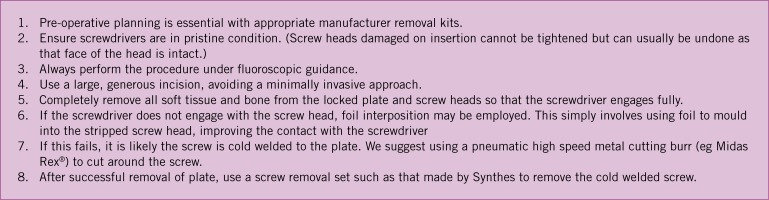
Proposed guidelines for removal of locked plates

We recommend the formulation of local guidelines regarding implant removal in every orthopaedic and trauma unit. We have devised a protocol aimed at improving the method of locked plate removal (Fig 2). Further research on the disadvantages of retained hardware and the complications of implant removal is also required.
